# At 4.5 but not 5.5 years, children favor kin when the stakes are moderately high

**DOI:** 10.1371/journal.pone.0202507

**Published:** 2018-08-16

**Authors:** Annie C. Spokes, Elizabeth S. Spelke

**Affiliations:** Department of Psychology, Harvard University, Cambridge, Massachusetts, United States of America; Swinburne University of Technology, AUSTRALIA

## Abstract

Adults report more willingness to help siblings over close friends when the stakes are extremely high, such as when deciding whether to donate a kidney or risk injury to rescue someone in peril. When dividing plentiful, low-value resources, in contrast, children expect people to share equally with friends and siblings. Even when distributing limited resources—one instead of many—and distributing to their own social partners rather than fictional characters, children share more with kin and friends than with strangers but do not favor kin over friends until 5.5 years of age. However, no study has tested whether children would preferentially benefit kin if the rewards require that children incur a higher personal cost of their own time and effort. In the present experiment, therefore, we asked if children would work harder for kin over non-kin when playing a challenging geometry game that allowed them to earn rewards for others. We found that 4.5-year-old children calibrated their time and effort in the game differently according to who received the rewards—they played for more trials and answered more trials correctly for kin over non-kin, but 5.5-year-old children did not. The older children may have found the task easier and less costly or may have different social experiences affecting their efforts to benefit others. Nonetheless, 4.5-year-old children’s social decisions favored kin as recipients of their generosity.

## Introduction

If we look across the animal kingdom, we see plenty of examples of species who have mechanisms for helping, protecting, and otherwise benefitting their kin over non-kin: from tiny social insects to birds and primates [1–7]. For example, Belding’s ground squirrels (*Spermophilu beldingi*) announce a predator with an alarm call at higher rates to benefit their squirrel relatives than non-relatives [8]. In many animals, genetic relatedness correlates positively with altruism and negatively with certain forms of violence [4,9]. Here we ask how do humans fit into this picture, as children transition from the preschool to the primary school years.

Evolutionary biologists and psychologists posit that kin relations influence human adults’ decisions in a social transaction, such that relatedness tracks with observable social behaviors—being more related to someone should increase positive behaviors like sharing and decrease negative behaviors like stealing [1,10–13]. Indeed, people give more to relatives than non-relatives [14], and signals to sibling relatedness increase reported altruism and aversion to sibling incest [15–16]. In an experimental paradigm that gave people the opportunity to win money for others by taking a personal cost and holding a challenging “wall-sit” physical position, with time linked to earnings, people held the position for longer to win more money for those more closely related to them—holding longer for self than a sibling or parent and longer for sibling and parent than a grandparent, aunt, uncle, niece or nephew [17]. They also worked harder for close kin—siblings or parents—than non-kin—friends or a charity [17]. Thus, kinship does hold value in the social currency of adults, though like other species—e.g., chimpanzees [18]—adults also benefit people outside kin relationships: friends, co-workers, and even one-shot interaction partners [14], [17,19]. Cooperation with these non-kin individuals is thought to be driven by principles of direct and indirect reciprocity [12,19]. However, a kinship preference is distinct from other relationships in that it is most present and pronounced in cases when the personal costs are high. When donating an organ or risking injury to save the life of another person, adults report more willingness to help siblings over close friends [20]. Increasing the stakes decreases the likelihood of altruism for friends or acquaintances but not kin [14,21–24].

How do humans develop this predisposition? A bias to favor kin could be something adults learn through culture or social experience, but the presence of kin-benefitting mechanisms across species suggest that kin over non-kin preference may be present in childhood and even infancy. The present experiment tests whether children demonstrate a kin preference when benefiting others in a situation that requires higher personal cost.

Children demonstrate some sensitivity to the recipient of the resources: they hold friends in higher regard than enemies [25–26], and friends and siblings in higher regard than strangers [27–28]. When children witness unequal resource distribution in a third-party context, they infer that the children favored others who were “friends” or who “liked each other more” [29]. However, young children expect equal sharing between siblings and friends, even when the resources are plentiful [27] or limited to one item [28]. Moreover, when children are divvying out significant rewards to their own social partners—their own siblings and friends—rather than fictional characters, children tend to share equally between them [28].

One possible reason for young children’s failure to favor siblings over friends might stem from their limited understanding of these kinship terms. In one study, 4.5- and 5.5-year-old children were asked factual questions about the social relations between individuals described as “brothers/sisters,” “friends,” or “strangers.” Children at 5.5 years answered all the questions correctly but 4.5-year-old children answered many questions at random, suggesting interesting developmental differences in children’s explicit, verbal understanding of friendship and kinship [28]. In contrast, 3.5- and 4.5-year-old children selectively shared with a friend over a stranger [28], showing that they understood the sharing questions and responded consistently. Curiously, 5.5-year-old children did not selectively share with friends over strangers.

Preschool children’s equal division of rewards to siblings and friends in previous studies could have occurred for two reasons. First, young children simply may not favor kin over non-kin when comparing siblings and friends no matter the situation: the high-stakes kinship preference present later in adulthood may result from learning and experience [15]. Second, the hypothetical costs and rewards used may not have been relevant or valuable to children. Children did not have to pay any personal cost in the previous scenarios [27–28]. To distinguish between these hypotheses, we asked whether children would show a preference for kin over non-kin if the cost was more relevant to and demanding of them—their own time and effort.

Even from a young age, infants and children track the costs of actions. Infants expect animated characters to prefer a character that they took a greater personal cost to reach [30]. Young children also infer that taking a higher cost to obtain something—like a particular snack—means the person likes that snack more [31]. Their early understanding of how costs affect others’ social choices suggest that children could attune their own behaviors to flexibly adapt their effort differently for targets they value more—potentially, kin over non-kin.

In our experiment, the action cost on which we focused concerned their own time and effort. In this experiment, children played a challenging geometry game that became more difficult with each trial (from [32–33]). In each round, they could earn stickers for a different recipient: themselves, a parent, sibling, friend, or an unfamiliar child. Children could end the round whenever they wanted and were asked after each trial whether they would like to stop or continue. First, children played for themselves, with feedback on their accuracy after each trial, and they ultimately received their payout of earned stickers. Children then played two more rounds of the game to win stickers for other people, without feedback on their accuracy.

We predicted that 4.5-year-old children would demonstrate a kinship preference because the opportunity to benefit others with stickers required children to take a personal cost of their own time and effort, raising the stakes of the situation. Thus, we predicted that children would work harder and persist for longer in the task for kin—parents and siblings—over non-kin—friends and strangers. To quantify their effort, we measured the number of trials that the children chose to play, the number of trials that children answered correctly, and the duration of their game play. We predicted that all of these measures should reflect more effort for kin than non-kin: playing more trials, answering more trials correctly, and playing for longer. This pattern of responses would suggest that children, like adults, do show a kinship preference in contexts that are higher stakes or require more personal cost to benefit the recipient. We also conducted exploratory analyses testing for differential playing to benefit specific recipients.

We also tested children at 5.5 years of age, with three possible hypotheses for their performance relative to 4.5-year-old children. First, older children may show a weaker kin preference than younger children. As noted above, 5.5-year-old children failed to favor friends over strangers in past research using low-stakes helping actions, in contrast to 4.5-year-old children [28]. Second, older children may show a stronger kin preference than younger children, with the hypothesis that they are learning from social experiences and observing the behavior of their relatives and friends. Lastly, there may be no difference between the two age groups.

## Materials and methods

### Participants

Forty-eight children from the Cambridge and Boston area participated in this study, with 24 children in each of two age groups centering on 4.5 years (12 female, 48.4–59.17 months, mean age = 53.05 months) and 5.5 years (12 female, 60.1–70.57, mean age = 66.09 months). Twelve children in each age group played for each of the four possible recipients (parent, sibling, friend, or unfamiliar child). Children were assigned to one of the counterbalanced orders and played for one kin and one non-kin recipient, two kin recipients, or two non-kin recipients. Two children were excluded from the study for not completing it (1) or for parental interference (1). After the study, children received a gift, and parents were reimbursed for their travel. This study was reviewed and approved by the Committee on the Use of Human Subjects in Research at Harvard University, and written informed consent was obtained from a parent or legal guardian of all subjects as well as verbal agreement from participants.

### Materials

**Fig 1:**
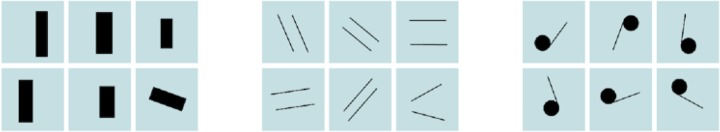
Sample images from the visual form analysis task.

Children completed three rounds of a visual form analysis task on a laptop computer adapted from [32] (Fig 1 and S1 Fig). The experimenter presented children with a visual array that had six 2D shapes: five similar shapes and one deviant shape that differed in one of a variety of geometric properties, including global shape, proportional length, angle size, symmetry, relations of parallelism and alignment, rotation, and sense relations.

Children had the opportunity to win stickers and could chose sticker type from over 20 different sticker types. The experimenter wrote each sticker recipient’s name on an envelope and put any stickers won in the envelope after each round, including an envelope for the participant.

### Procedure

Children played three rounds of a geometry game on the computer to win stickers first for themselves and then for two out of four assigned recipients, with the exception that children who did not have siblings were not assigned to play for a sibling. One experimenter played the first round with children and instructed them on their two recipients (parent, sibling, friend, or unfamiliar child), and a second experimenter—unaware of who children were playing for—played the second two rounds with children. Each round continued until children chose to stop playing or finished all possible trials.

Children were invited to play a visual form analysis task on the computer in a quiet testing room in the Laboratory for Developmental Studies, and sessions were recorded on video. Children were shown a visual array with six 2D shapes and asked to look carefully at all of the shapes and point to the one that did not belong with the rest. Trials increased in difficulty based on similar aged children’s performance on the task in prior research [32–33]. After the child pointed to his or her choice for the deviant shape, an experimenter asked the child if he or she would like to keep playing or stop playing. If the child chose to keep playing, the experimenter selected the associated key on the keyboard to record the child’s choice and advanced to the next trial. If the child chose to stop playing, the experimenter selected the associated key and then closed the laptop to end that round.

First, one experimenter introduced the game and walked children through two practice trials with feedback on their responses. Next, children were instructed that for each trial they answered correctly, they earned one sticker and got to choose whether to keep playing or stop playing. The experimenter also told the child that the trials got harder as they continued. Children then played up to ten trials with feedback after each trial and tracking of their total correct. After children chose to stop playing or completed ten trials, they selected their prize stickers and put them in an envelope with their names on it.

Next, the first experimenter explained that the children would play the game two more times and win stickers for other people. The experimenter told the children that they would play for a: sibling, parent, friend, or child they have never met before (i.e., “stranger”). When playing for a sibling, parent, or friend, the experimenter then encouraged the children to name a specific person that fit in that relationship, and the experimenter wrote that person’s name on an envelope. For the stranger, the experimenter had already written a different child’s name (gender matched to the participant) on the envelope prior to the experiment and explained that that child would come into the lab another time to collect the stickers. The experimenter repeated this for a second recipient. The order of recipient was counterbalanced across participants.

The first experimenter told the children that a new experimenter (who they had met previously in the lobby) would play the next round of the game with them. This experimenter was unaware of the recipients, and the first experimenter encouraged children not to share the recipient identity with the second experimenter by telling them it was a surprise that would be revealed to the second experimenter at the end of the game. The first experimenter reminded the children who they were playing for on that round and then left the envelopes facedown and left the room. Thus, the second experimenter was unaware of the type of recipient for whom children were playing on any given trial.

For the second and third rounds, children played the visual form analysis task with the second experimenter and could play up to 18 trials, with increasing difficulty. The experimenter did not give any feedback and asked after each trial whether the children wanted to keep playing or stop playing.

Children took a break between the second and third rounds, during which they had the opportunity to color or read a short storybook with the first experimenter. Before starting the third round, the first experimenter reminded children who the recipient was for that round before leaving the room so the second experimenter could conduct the visual form analysis task.

After children completed the second and third rounds, they chose stickers to distribute to each of the recipients based on a rough estimate of how many trials they had completed.

We predicted that children would take a greater personal cost by continuing with the challenging task for longer and trying to win more stickers for kin (parents and siblings) over non-kin (friends and strangers). To capture their effort, we measured the number of trials played, the number of trials answered correctly, and the duration of play. We predicted that children would play longer, for more trials, and more accurately for kin over non-kin, demonstrating they invested more of their time and effort into the task when benefiting kin. Trial completion and accuracy were recorded in the visual form analysis task software and confirmed from video. The duration of play was coded from the video from the start of the first trial when the child asked to stop playing or the 18^th^ trial was completed.

## Results

### Younger children

**Fig 2:**
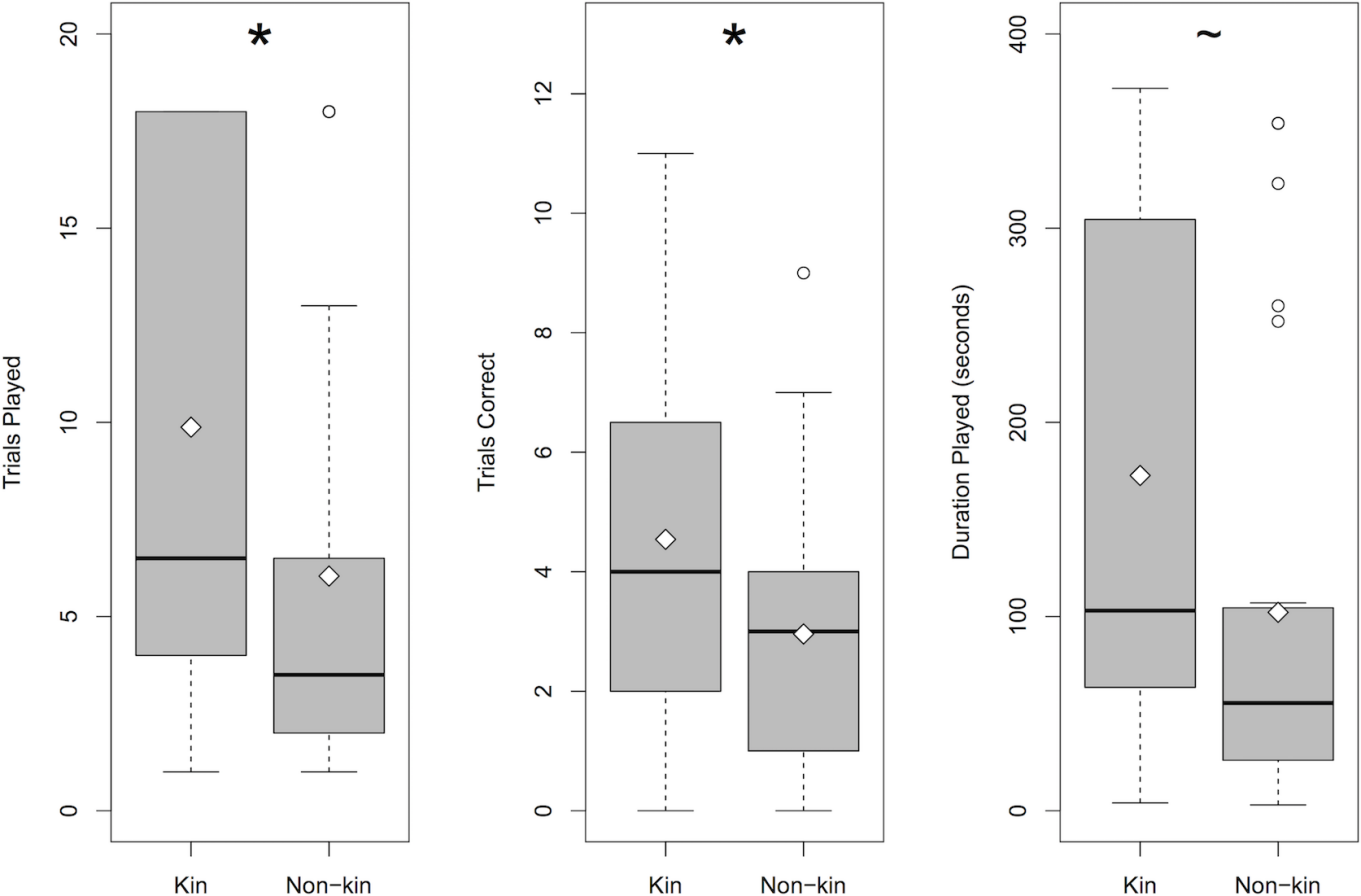
Children’s performance in the visual form analysis task at 4.5 years. (A) Children (*n* = 24) played more trials for kin than non-kin, (B) answered correctly on more trials, and (C) played the game for marginally longer (*P < 0.05, ~*P* = 0.083).

For children at 4.5 years, data for the number of geometry trials and duration played were log-normally distributed, so we log transformed these variables prior to analyses [34]. Across these measures, one-way ANOVAs testing for a difference in how children played for kin versus non-kin revealed that children (*N* = 24) played more geometry trials for their kin relations—siblings and parents—than for non-kin—friends and strangers, *F*(1, 46) = 4.27, *p* = .044. These children also answered more geometry trials correctly, *F*(1, 46) = 4.57, *p* = .038, and played marginally longer, *F*(1, 46) = 3.14, *p* = .083, for kin than for non-kin (Fig 2). In exploratory one-way ANOVAs testing all four recipient categories, there was no main effect of recipient for number of geometry trials played, *F*(3, 44) = 1.51, *p* = 0.23, number of geometry trials answered correctly, *F*(3, 44) = 1.68, *p* = 0.19, and duration of geometry play, *F*(3, 44) = 1.039, *p* = 0.39. In exploratory pairwise comparisons using paired *t*-tests and a Holm correction for multiple comparisons, children did not show significant differences for any pair of recipients for geometry trials played (*p*s > 0.44), geometry trials answered correctly (*p*s > 0.29), and duration played (*p*s > 0.95). Exploratory analyses using two-way ANOVAs revealed a main effect of children’s gender with females playing more geometry trials, *F*(1, 44) = 6.97, *p* = 0.011, and for a longer duration than males, *F*(1, 44) = 5.89, *p* = 0.019, but there was no main effect of children’s gender on the number of geometry trials answered correctly, *F*(1, 44) = 1.004, *p* = 0.32, and there were no interactions between children’s gender and kin versus non-kin recipient on any of the three measures (*p*s = 0.51, 0.52, 0.82, respectively).

### Older children

**Fig 3:**
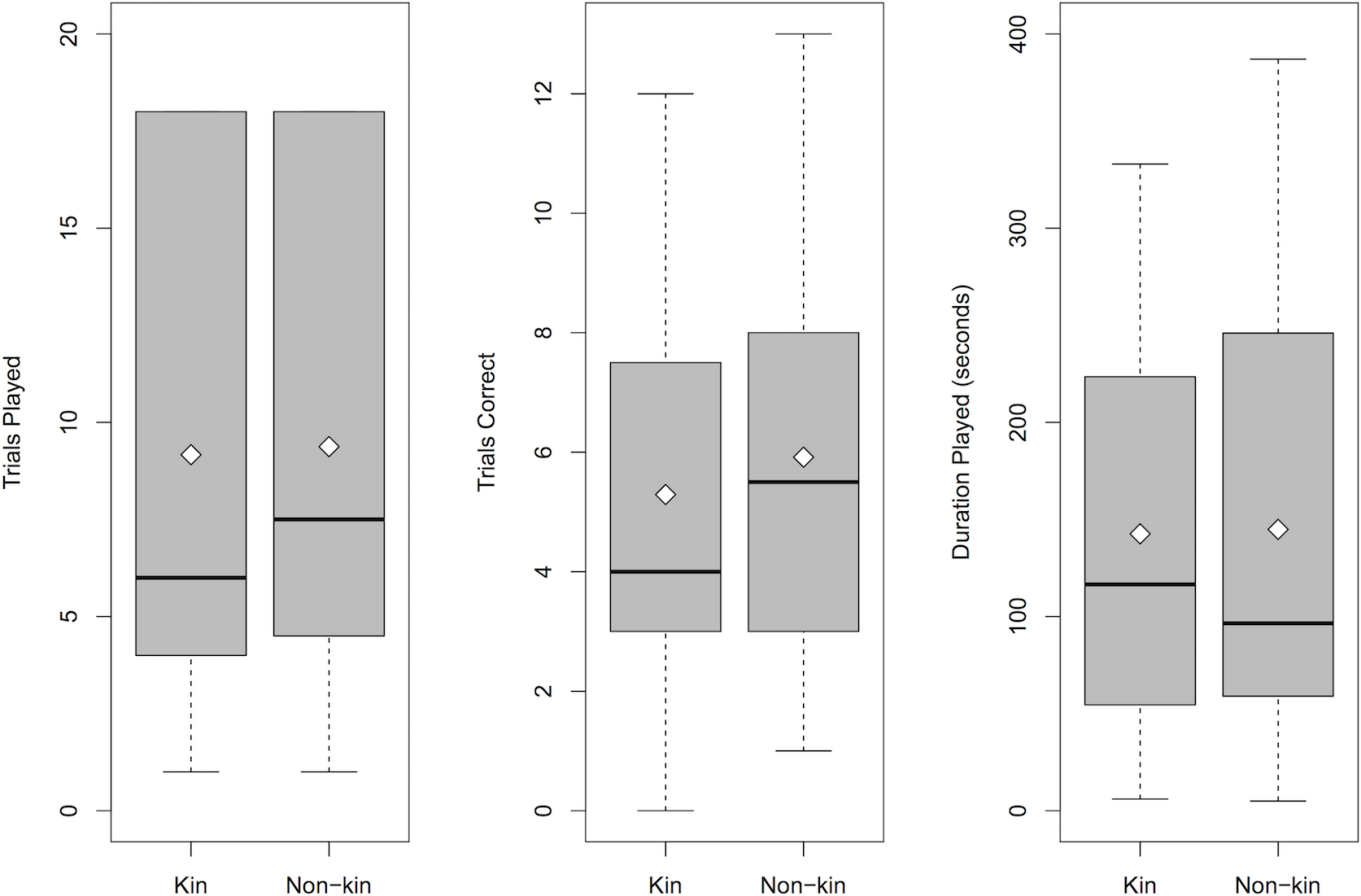
Children’s performance in the visual form analysis task at 5.5 years. Children’s (*n* = 24) performance in the visual form analysis task, as measured by (A) number of trials played for kin and non-kin, (B) number of trials answered correctly for kin versus non-kin, and (C) duration of play for kin versus non-kin.

For children at 5.5 years, one-way ANOVAs testing for a difference in how children played for kin versus non-kin revealed that children (*N* = 24) did not differ when playing for kin versus non-kin in the number of geometry trials played, *F*(1, 46) = 0.013, *p* = 0.91, the number of geometry trials answered correctly, *F*(1, 46) = 0.42, *p* = 0.52, or the duration of geometry game play, *F*(1, 46) = 0.005, *p* = 0.94 (Fig 3). In exploratory one-way ANOVAs testing all four recipient categories, there was no main effect of recipient for geometry trials played, *F*(3, 44) = 0.22, *p* = 0.88, geometry trials answered correctly, *F*(3, 44) = 0.26, *p* = 0.86, and duration played, *F*(3, 44) = 0.16, *p* = 0.92. In exploratory pairwise comparisons using paired *t*-tests and a Holm correction for multiple comparisons, children did not show significant differences for any pair of recipients in geometry trials played (*p*s > 1), geometry trials answered correctly (*p*s > 1), and duration played (*p*s > 1), and these patterns of results were the same for log transformed and raw data. Exploratory analyses using two-way ANOVAs revealed no main effects of children’s gender on the number of geometry trials played (*p* = 0.73), geometry trials answered correctly (*p* = 0.68), or duration of geometry play (*p* = 0.39). In addition, there were no interactions between children’s gender and kin versus non-kin recipient on any of the three measures (*p* = 0.73, *p* = 0.43, *p* = 0.91, respectively).

### Comparing age groups

Exploratory analyses comparing younger and older children to one another using two (recipient: kin versus non-kin) by two (age group: 4.5- and 5.5-year-olds) ANOVAs for each of the three dependent variables revealed no significant main effects or interactions. For geometry trials played, there was a marginal age group by recipient interaction, *F*(1, 92) = 3.13, *p* = 0.08, but no main effect of age group, *F*(1, 92) = 2.34, *p* = 0.13, or recipient, *F*(1, 92) = 1.77, *p* = 0.19. For geometry trials answered correctly, there was a marginal effect of age group, *F*(1, 92) = 3.48, *p* = 0.066, with 5.5-year-old children answering more trials correctly, and no effect of recipient, *F*(1, 92) = 0.39, *p* = 0.53, or recipient by age group interaction, *F*(1, 92) = 2.59, *p* = 0.11. For duration of geometry play, there were no significant main effects (recipient: *F*(1, 92) = 1.52, *p* = 0.22; age group: *F*(1, 92) = 1.37, *p* = 0.25) or interaction, *F*(1, 92) = 2.39, *p* = 0.13.

## General discussion

We found that 4.5-year-old children played more geometry trials and answered more geometry trials correctly for kin than non-kin, and their duration of geometry play was consistent with this pattern. These findings provide the first evidence that young children do favor kin, in circumstances that require them to take a personal cost to earn rewards for the recipient. Children took their time and energy to continue with a challenging geometry game to potentially win stickers for someone else, when alternatively, they could finish the game and be doing something more enjoyable or beneficial for themselves. This requirement of personal costs thus may have increased the stakes higher than in previous studies in which children distributed windfall resources to unfamiliar and familiar recipients [27–28].

On the hypothesis that kin favoritism develops in response to social experience, 5.5-year-old children should have shown the same or a stronger pattern of kin preference, but the findings did not support t his hypothesis. Based on past research, we suggest that two factors may explain the failure of the older children to work harder for kin. First, the geometry game we used has revealed consistent effects of age in past research [33], so children may have found the task easier, and therefore less costly, at the older age. Consistent with this possibility, older children showed a marginal tendency to answer more geometry trials correctly, even though they did not either attempt more trials or play for a longer duration than younger children. Second, most 5.5-year-old children attend school, where they likely are encouraged to befriend same aged children they have never met before—like the unknown target children in our study. Fairness norms may also be more salient, expected, and enforced in school-aged children [35]. Consistent with this possibility, 6-year-old children pay a personal cost to punish others who do not follow fairness and equality norms in sharing [36–37]. Future research that tests young school-aged children with a more difficult task and that tracks their school experience could shed light on the factors that modulate 5.5-year-old children’s choice to take more versus less costly actions for kin over non-kin.

The present experiment adds to building evidence that children reward, help, and share with others who are socially closer to them (e.g., [25–28]), and provides new, albeit mixed, evidence that young children are sensitive to kinship. Four-year-old children calibrated their time and effort in a task differently according to who would reap the rewards when the stakes were higher and the necessary costs—in their own time and effort—were meaningful to them. Thus, children may be similar to adults in their consideration of kin in social decisions, showing a more distinct kin bias in situations involving high personal cost [14]. However, we did not find evidence that children favor kin over non-kin in our exploratory analyses directly comparing different recipient types (e.g., siblings versus friends), possibly because our experiment was underpowered to detect such effects. Future research could further explore these direct comparisons with a larger sample of children.

Contrary to our hypothesis of an early-emerging kinship bias, explicit reasoning about kinship appears to be slow to emerge over the early childhood years. When asked explicitly about distinctions between siblings, friends, and strangers, children do not demonstrate clear understanding of kinship relations until around 5.5 years of age [28], and they do not demonstrate knowledge of the biology underlying blood relations or definitions of kinship terms like “grandmother” until as late as seven years of age [38–39]. This slow-developing explicit knowledge of kinship is especially interesting given infants’ early sensitivity to kin-like caregiving relationships: infants are sensitive to caregiving networks even in their second year, expecting adults who respond to the same baby’s cries to affiliate with one another, and also expecting babies who are soothed by the same adult to affiliate with one another [40]. In addition to slow-developing kinship knowledge, children’s non-kin friendships may be more relevant and valuable in modern social environments, and children may be more sensitive to benefitting these non-kin reciprocal partners than we anticipated.

The present experiment presents a new experimental paradigm that could be used to explore further the nuanced social decisions of children who devote their personal time and energy to benefitting others. Research with larger samples of children playing for each recipient could better test for differences between recipients—do children work harder for siblings than friends? The present paradigm can also be used to track whether children are sensitive to different degrees of relatedness: would they work harder for a parent or sibling than a grandparent, aunt, uncle, niece, or nephew, as adults do when holding a wall-sit position [17]?

In summary, although animals from tiny social insects to non-human primates selectively aid kin over non-kin, this tendency appears to be diluted in young children. Although a tendency to favor kin modulates the choices of 4.5-year-old children, this tendency is not strong at that age, and it is not present a year later, at least in the situations that we have tested. This complex developmental pattern invites further study of children just before and after the start of primary school.

## Supporting information

S1 Fig Visual form analysis task images. All images in trial order for visual form analysis trials in practice and test.
(PDF)

S2 Fig Children’s performance in the visual form analysis task by recipient. (A) 4.5-year-old children (*n* = 24) played more trials for kin (sibling, parent) than non-kin (friend, stranger), (B) answered correctly on more trials, and (C) played the game for marginally longer (*P < 0.05, ~*P* = 0.083). (D) 5.5-year-old children (*n* = 24) played roughly the same number of trials for kin (sibling, parent) and non-kin (friend, stranger), (E) answered correctly on similar number of trials, and (F) played the game for a similar amount of time.
(TIF)

S1 Table Children’s performance in the visual form analysis task. 4.5- and 5.5-year-old children’s mean (and standard deviation) performance in the visual form analysis task when playing for a sibling, parent, friend, or stranger.
(PDF)
