# Supplementary material for: At 4.5 but not 5.5 years, children favor kin when the stakes are moderately high
Source: PLoS One. 2018 Aug 16;13(8):e0202507. doi: 10.1371/journal.pone.0202507 (PMC6095549; doi:10.1371/journal.pone.0202507)
Supplement: S1 Fig — All images in trial order for visual form analysis trials in practice and test. (PDF) [file pone.0202507.s001.pdf]

PRACTICE

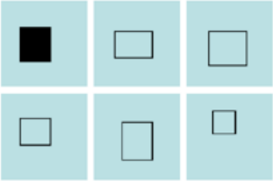
Trial 1

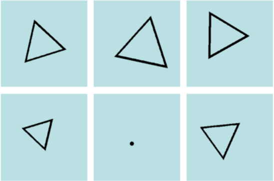
Trial 2

BLOCK 1 – Recipient: Self

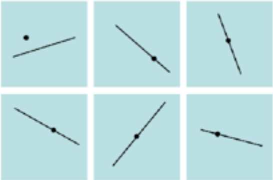
Trial 1

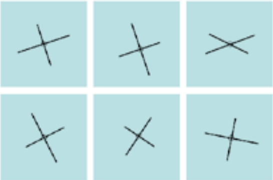
Trial 2

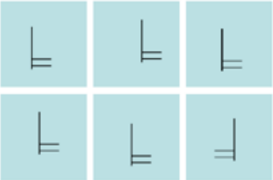
Trial 3

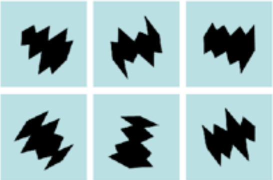
Trial 4

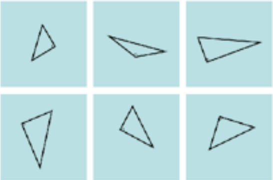
Trial 5

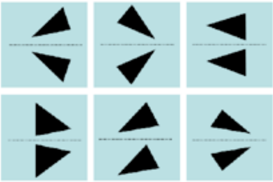
Trial 6

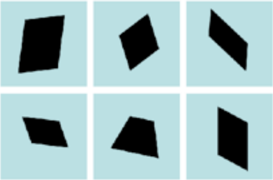
Trial 7

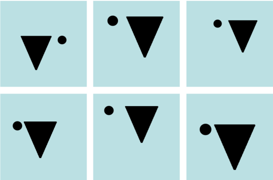
Trial 8

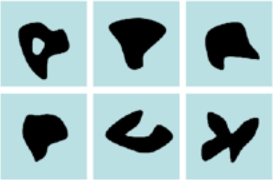
Trial 9

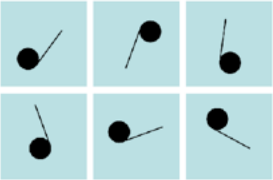
Trial 10

BLOCK 2 – Recipient: Sibling, Parent, Friend, or Stranger

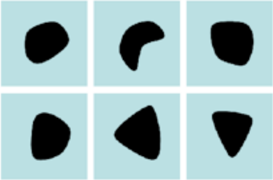
Trial 1

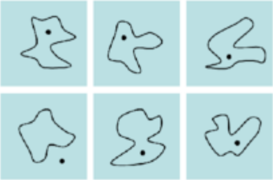
Trial 2

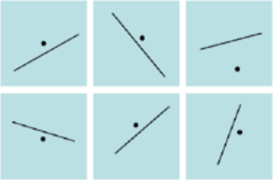
Trial 3

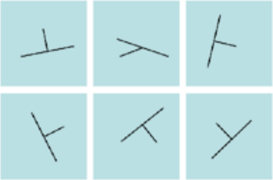
Trial 4

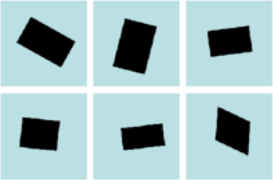
Trial 5

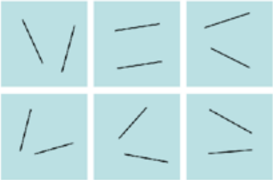
Trial 6

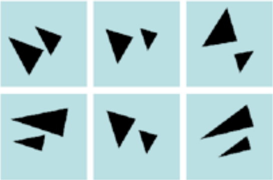
Trial 7

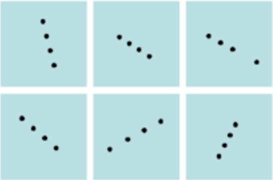
Trial 8

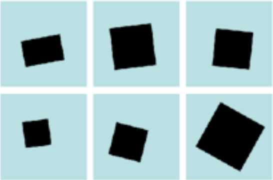
Trial 9

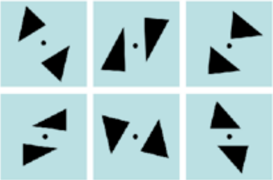
Trial 10

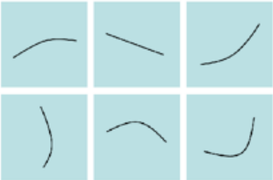
Trial 11

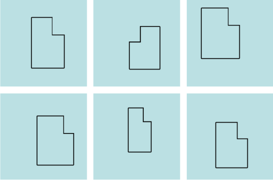
Trial 12

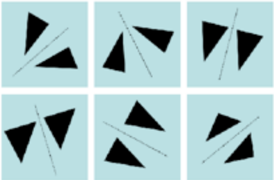
Trial 13

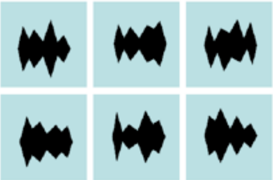
Trial 14

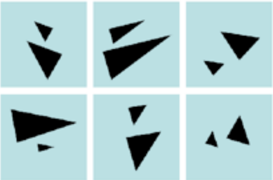
Trial 15

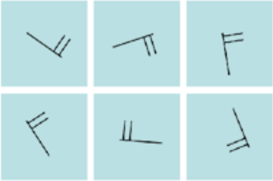

Trial 16

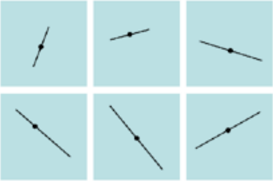

Trial 17

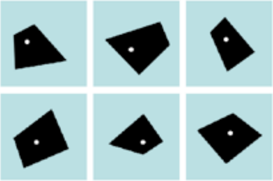

Trial 18

BLOCK 3 – Recipient: Sibling, Parent, Friend, or Stranger

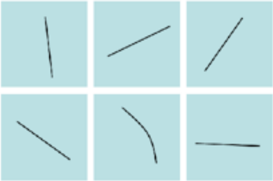

Trial 1

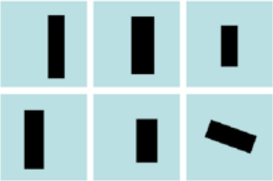

Trial 2

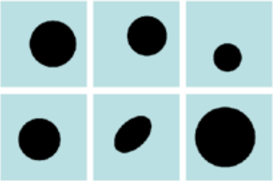

Trial 3

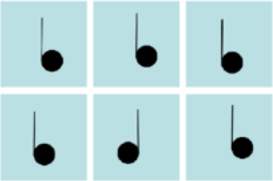

Trial 4

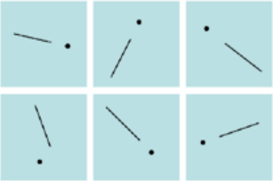

Trial 5

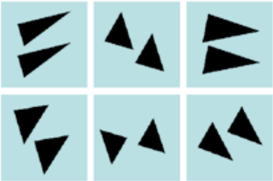

Trial 6

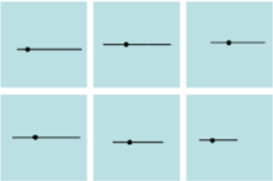

Trial 7

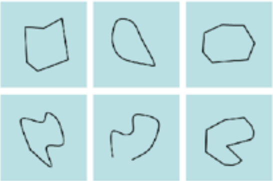

Trial 8

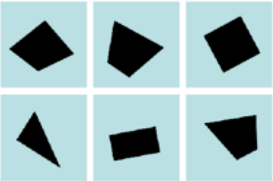

Trial 9

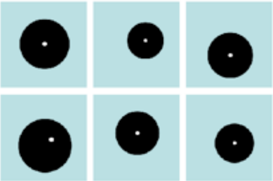

Trial 10

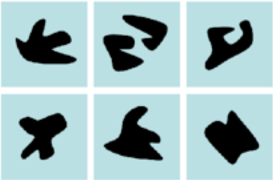

Trial 11

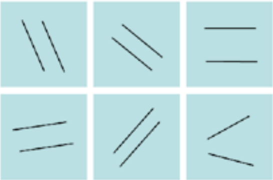

Trial 12

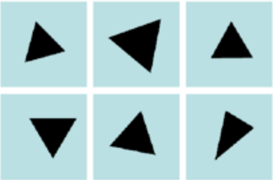

Trial 13

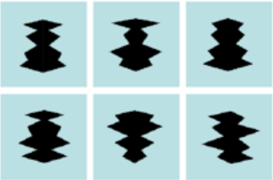

Trial 14

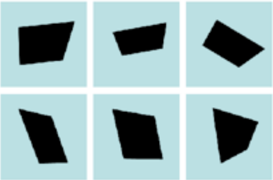

Trial 15

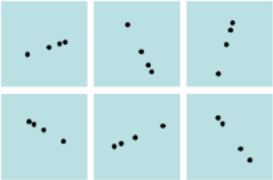

Trial 16

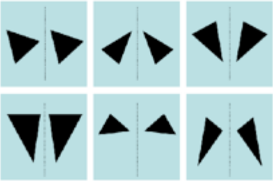

Trial 17

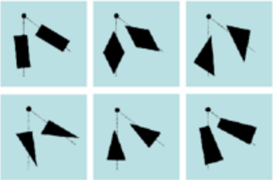

Trial 18
