# Supplementary figures and images for: At 4.5 but not 5.5 years, children favor kin when the stakes are moderately high
Source: PLoS One. 2018 Aug 16;13(8):e0202507. doi: 10.1371/journal.pone.0202507 (PMC6095549; doi:10.1371/journal.pone.0202507)

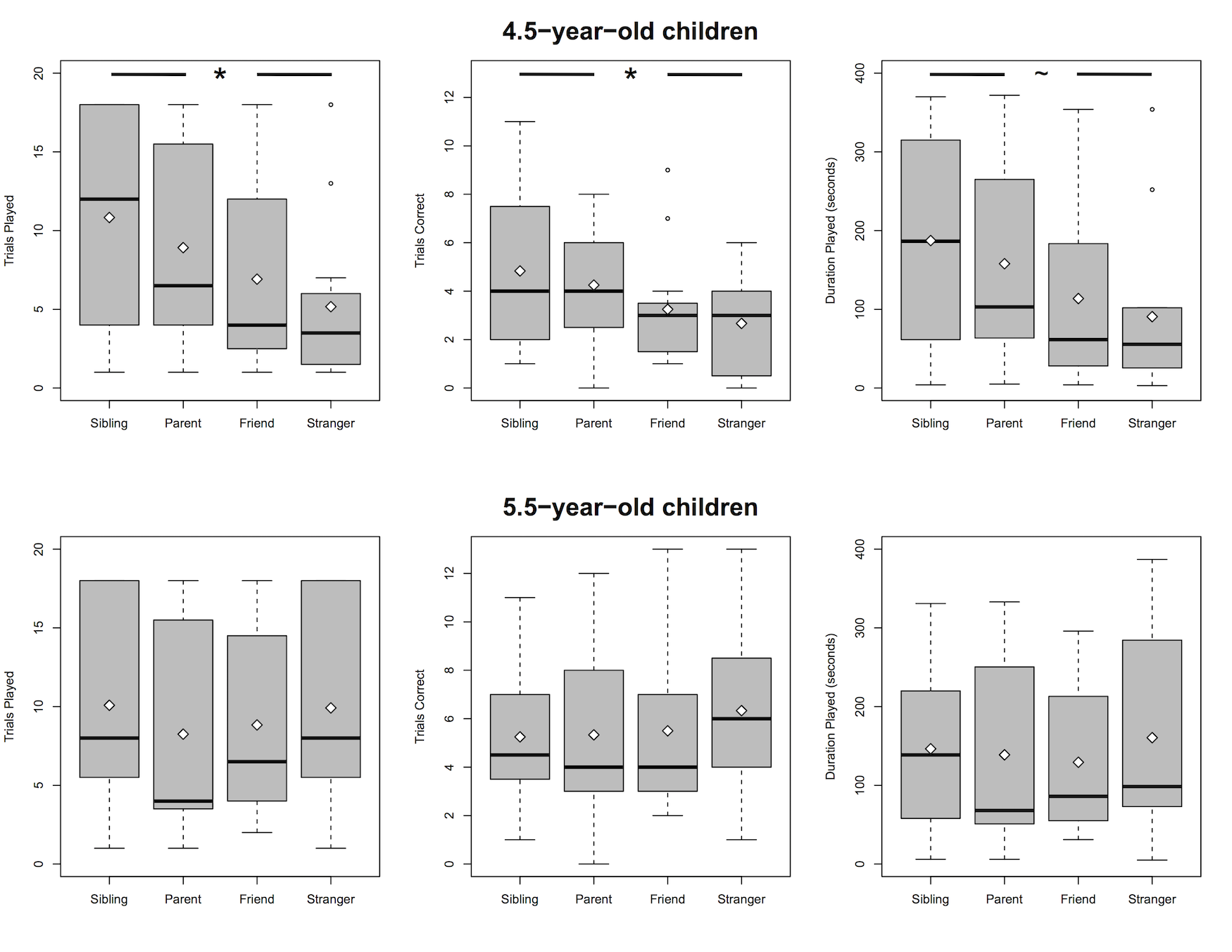

Supplement: S2 Fig — (A) 4.5-year-old children (n = 24) played more trials for kin (sibling, parent) than non-kin (friend, stranger), (B) answered correctly on more trials, and (C) played the game for marginally longer (*P < 0.05, ~P = 0.083). (D) 5.5-year-old children (n = 24) played roughly the same number of trials for kin (sibling, parent) and non-kin (friend, stranger), (E) answered correctly on similar number of trials, and (F) played the game for a similar amount of time. (TIF) [file pone.0202507.s002.tif]
