# Supplementary material for: At 4.5 but not 5.5 years, children favor kin when the stakes are moderately high
Source: PLoS One. 2018 Aug 16;13(8):e0202507. doi: 10.1371/journal.pone.0202507 (PMC6095549; doi:10.1371/journal.pone.0202507)
Supplement: S1 Table — 4.5- and 5.5-year-old children’s mean (and standard deviation) performance in the visual form analysis task when playing for a sibling, parent, friend, or stranger. (PDF) [file pone.0202507.s003.pdf]

S1 Table. Children's performance in the visual form analysis task.

		Sibling	Parent	Friend	Stranger
**4.5-year-olds**	**Trials Played**	10.83 (7.65)	8.92 (6.32)	6.92 (6.86)	5.17 (5.25)
	**Trials Correct**	4.83 (3.43)	4.25 (2.3)	3.25 (2.45)	2.67 (2.02)
	**Duration (Min.)**	3.12 (2.44)	2.63 (2.05)	1.89 (2.09)	1.51 (1.77)
**5.5-year-olds**	**Trials Played**	10.08 (6.49)	8.25 (6.76)	8.83 (6.11)	9.92 (6.52)
	**Trials Correct**	5.25 (2.83)	5.33 (3.63)	5.5 (3.5)	6.33 (3.55)
	**Duration (Min.)**	2.44 (1.68)	2.31 (2.03)	2.15 (1.59)	2.68 (2.17)

4.5- and 5.5-year-old children's mean (and standard deviation) performance in the visual form analysis task when playing for a sibling, parent, friend, or stranger.
